# LAP TGF-Beta Subset of CD4^+^CD25^+^CD127^−^ Treg Cells is Increased and Overexpresses LAP TGF-Beta in Lung Adenocarcinoma Patients

**DOI:** 10.1155/2015/430943

**Published:** 2015-10-25

**Authors:** Lorenzo Islas-Vazquez, Heriberto Prado-Garcia, Dolores Aguilar-Cazares, Manuel Meneses-Flores, Miriam Galicia-Velasco, Susana Romero-Garcia, Catalina Camacho-Mendoza, Jose Sullivan Lopez-Gonzalez

**Affiliations:** ^1^Departamento de Enfermedades Cronico-Degenerativas, Instituto Nacional de Enfermedades Respiratorias Ismael Cosio Villegas, Tlalpan 4502, Colonia Seccion XVI, 14080 Mexico, DF, Mexico; ^2^Posgrado en Ciencias Biologicas, Universidad Nacional Autonoma de Mexico, Avenida Ciudad Universitaria 3000, 04510 Mexico, DF, Mexico; ^3^Clinica de Neumo-Oncologia, Instituto Nacional de Enfermedades Respiratorias Ismael Cosio Villegas, Tlalpan 4502, Colonia Seccion XVI, 14080 Mexico, DF, Mexico

## Abstract

Lung cancer is the leading cause of cancer death worldwide. Adenocarcinoma, the most commonly diagnosed histologic type of lung cancer, is associated with smoking. Cigarette smoke promotes inflammation on the airways, which might be mediated by Th17 cells. This inflammatory environment may contribute to tumor development. In contrast, some reports indicate that tumors may induce immunosuppressive Treg cells to dampen immune reactivity, supporting tumor growth and progression. Thus, we aimed to analyze whether chronic inflammation or immunosuppression predominates at the systemic level in lung adenocarcinoma patients, and several cytokines and Th17 and Treg cells were studied. Higher proportions of IL-17-producing CD4^+^ T-cells were found in smoking control subjects and in lung adenocarcinoma patients compared to nonsmoking control subjects. In addition, lung adenocarcinoma patients increased both plasma concentrations of IL-2, IL-4, IL-6, and IL-10, and proportions of Latency Associated Peptide (LAP) TGF-*β* subset of CD4^+^CD25^+^CD127^−^ Treg cells, which overexpressed LAP TGF-*β*. This knowledge may lead to the development of immunotherapies that could inhibit the suppressor activity mediated by the LAP TGF-*β* subset of CD4^+^CD25^+^CD127^−^ Treg cells to promote reactivity of immune cells against lung adenocarcinoma cells.

## 1. Introduction

Lung cancer is the leading cause of cancer death worldwide. Non-small cell lung carcinoma (NSCLC) is the most common type of lung cancer. Adenocarcinoma is the most frequently diagnosed histologic type of NSCLC and is associated with passive and active smoking. The substantial doses of carcinogens and toxins contained in cigarette smoke favor chronic inflammation of the respiratory tract, which is a risk factor for the development of nonmalignant and malignant diseases [1]. Currently, accumulating evidence has shown that inflammation is associated with the pathogenesis of lung cancer, especially inflammation induced by cigarette smoke [2, 3]. Several authors have proposed that tumor cells induce and maintain an inflammatory reaction. A tumor-associated inflammatory response can contribute to multiple capacities associated with the development and progression of cancers [4–6].

In chronic inflammation, the participation of the Th17 cell subpopulation is of primary importance. Th17 cells are induced by transforming growth factor beta (TGF-*β*) and interleukin- (IL-) 6 or IL-23. These cells express the retinoic orphan receptor gamma (ROR-*γ*t) and present a potent proinflammatory activity, which is mediated predominantly by their effector cytokines IL-17A/F, IL-21, IL-22, and IL-23 [7]. In cancer, Th17 cells have been reported to show pro- and antitumoral effects. The protumoral role of Th17 cells is based on their capacity to recruit neutrophils through IL-8 production. In addition, IL-17A induces that stromal cells and fibroblast produce angiogenic factors such as VEGF. Also, IL-17 induces production of IL-6 by malignant cells, which activate Stat3 signaling pathway. However, Th17 cells might have an antitumoral activity mediated indirectly through dendritic cells (DCs) recruitment and activation of cytotoxic CD8^+^ T-cells activity [8–10].

On the other hand, tumor cells develop several strategies to hamper the activity of the distinct types of cells participating in the immune response, and there is evidence that regulatory T-cells (Treg cells) play an important role in suppressing antitumoral functions [11, 12]. Treg cells are a subset of CD4^+^ T-cells that are specialized for suppressive function. Treg cells differentiate from naïve CD4^+^ T-cells in presence of soluble factors such as IL-10 and TGF-*β* [13, 14]. The transcription factor FOXP3 has been shown to play a key role in regulatory T-cell function and is a characteristic marker for these cells [14]. However, FOXP3 is a nuclear protein that has a limited value in the isolation of Treg cells for functional assays. Recently, low levels of the IL-7 receptor *α*-chain (CD127) have been shown to be expressed on Treg cell surface and are inversely correlated with FOXP3 expression [15]. Thus, the more reliable phenotype for identifying Treg cells is CD4^+^CD25^+^CD127^−^.

Treg cells mediate immunosuppression via cytotoxic T-lymphocyte-associated protein 4 (CTLA-4), IL-10, and cell surface expression of TGF-*β* bounds to membrane through Latency Associated Peptide (LAP) [14, 16–19]. LAP is the N-terminal propeptide of the TGF-*β* precursor that noncovalently binds to TGF-*β*, forming a latent TGF-*β* complex and favoring the release of TGF-*β* into the extracellular milieu. Recently, a subset of inducible LAP^+^ Treg subset has been reported; this subset suppresses proliferation of conventional T-cells* in vitro *via IL-10 and TGF-*β* [20–22].

Several studies have shown that Th17 and Treg cells are found in peripheral blood of lung cancer patients [23, 24]; however, the possible interrelation between these subsets remains to be elucidated. The objective of the present study is to clarify to what extent smoking-associated chronic inflammation versus tumor induced suppression contributes in advanced-stage lung adenocarcinoma patients; thus, several cytokines, Th17, and Treg cells were quantified and compared with smoking and nonsmoking controls subjects. Our data indicate that cigarette smoke induced a proinflammatory profile; nevertheless, lung tumors favored suppression rather than inflammation and lead to increased levels of immunosuppressive cytokines and upregulation of LAP-TGF-*β* in the CD4^+^CD25^+^CD127^−^ Treg cells. This Treg cell subset might mediate the local and systemic suppression in lung adenocarcinoma patients. Targeting Th17/Treg balance for therapeutic purposes may represent a useful tool for lung cancer treatment in the future.

## 2. Materials and Methods

### 2.1. Population Studied

The population consisted of a total of 28 patients with clinical stage IV lung adenocarcinoma. The diagnosis was established according to WHO criteria [25] by histological examination of biopsy specimens or cytological observation of malignant cells in pleural effusion. Only patients who were classified as heavy smokers were included in the study. According to gender they were 16 males and 12 females. The median age of the group was 59 years (range = 41–78 years). None of the patients had received any type of anticancer therapy before the study.

As controls, 13 healthy nonsmoking (9 males and 4 females) and 15 heavy-smoking (10 males and 5 females) volunteers were included. The median age was 56 years in the nonsmoking group (range = 43–83 years) and 52 years in the smoking group (range = 45–63 years). Subjects from the control groups had normal values for lung function tests as measured by spirometry. The Committee of Science and Bioethics of the National Institute of Respiratory Diseases approved the protocol for the collection of biological samples. Written informed consent was obtained from each subject.

### 2.2. Plasma Collection and Isolation of Mononuclear Cells from Blood Samples

Blood samples in EDTA-containing tubes were centrifuged, and plasma was immediately collected and stored at −80°C. Peripheral blood mononuclear cells (PBMCs) were separated on Lymphoprep (Axis-Shield, Oslo, Norway) by centrifugation at 150 ×g for 45 min. Recuperated PBMCs were washed, suspended in freezing medium, and cryopreserved in liquid nitrogen.

### 2.3. Quantification of Plasma Th1, Th2, and Th17 Cytokines

Plasma IL-2, IL-4, IL-6, IL-10, IL-17A, TNF-*α*, and IFN-*γ* cytokines from lung adenocarcinoma patients and smoking and nonsmoking control subjects were measured simultaneously using the Cytometric Bead Array Human Th1/Th2/Th17 Cytokine kit (BD Biosciences, San Jose, CA, USA) according to the manufacturer's procedure. Data were analyzed using FCAP Array software version 1.0.1 (BD Biosciences).

### 2.4. Quantification of Plasma TGF-*β*1 by ELISA

TGF-*β*1 in plasma from the lung adenocarcinoma patients and smoking and nonsmoking control groups was quantified using the Quantikine Human TGF-*β*1 immunoassay (R&D system Inc., MN, USA) according to the manufacturer's procedure.

### 2.5. Percentage of IL-17A-Producing CD4^+^ T-Cells

PBMCs were cultured in Roswell Park Memorial Institute (RPMI) 1640 medium (Sigma Chemical, St. Louis, MO, USA) supplemented with 10% fetal calf serum and antibiotics (complete medium) and stimulated with bead particles coated with anti-CD2, anti-CD3, and anti-CD28 antibodies using the T-Cell Activation/Expansion kit (Miltenyi Biotec, Auburn, CA, USA) following the manufacturer's instructions for a period of 72 h. Five hours before completing the total incubation time, the PBMCs were further stimulated with phorbol 12-myristate 13-acetate (PMA) (25 ng/mL), ionomycin (1 *μ*g/mL), and brefeldin A (10 *μ*g/mL) (all from Sigma Aldrich, St. Louis, MO, USA). Cell stimulation was confirmed by membrane expression of CD69 molecule using an FITC anti-CD69 antibody (FN50 clone, BioLegend, San Diego, CA, USA) and flow cytometric analysis.

For intracellular detection of IL-17A, PBMCs previously stimulated and incubated with PE-Cy5 anti-CD4 antibody (RPA-T4 clone, BioLegend) were washed, permeabilized with FACS Permeabilizing Solution (BD Pharmingen), and fixed. Permeabilized cells were stained with PE anti-IL-17A (BL168 clone, BioLegend) and Alexa Fluor 488 anti-IFN-*γ* (4S.B3 clone, BioLegend) antibodies.

### 2.6. Purification of CD4^+^ T-Cells

CD4^+^ T-cells were isolated from PBMCs by negative selection with the CD4^+^ T-cell isolation kit II (Miltenyi Biotec, Auburn, CA, USA) following the manufacturer's instructions. Cell viability determined by trypan blue exclusion was always higher than 92%. The purity of the CD4^+^ T-cells was always higher than 94%, as detected by a mixture of FITC anti-CD3 (HIT3a clone), PE-Cy5 anti-CD8 (HIT8a clone), and PE anti-CD4 (RPA-T4 clone) antibodies (all from BioLegend) and flow cytometric analysis.

### 2.7. Th17 and Treg Phenotyping by Flow Cytometry

For Th17 cell immunostaining, purified CD4^+^ T-cells were identified using labeled monoclonal antibodies against the following molecules: Alexa Fluor 700 anti-CD4 and PE anti-ROR-*γ*t (AFKJS-9 clone, eBioscience, San Diego, CA, USA).

For Treg immunostaining, purified CD4^+^ T-cells were identified using labeled monoclonal antibodies against the following molecules: Alexa Fluor 700 anti-CD4 (RPA-T4 clone, BD Pharmingen), PE-Cy5 anti-CD25 (M-A251 clone, BD Pharmingen), Alexa Fluor 647 anti-CD127 (HCD127 clone, BioLegend), and Alexa Fluor 488 anti-FOXP3 (150D clone, BioLegend).

To analyze CTLA-4 expression in Treg cells, PE anti-CTLA-4 antibody (BNI3 clone, BD Pharmingen) was added. The integrated mean fluorescence intensity (iMFI) was calculated by multiplying the percentage of CTLA-4 by their corresponding mean fluorescence intensity (MFI), as described previously [26].

Intracellular staining for FOXP3 and ROR-*γ*t CD4^+^ T-cells was performed using a commercially available kit (BioLegend) following the manufacturer's instructions. Appropriate isotype controls were used to allow identification of positive and negative cell populations. To rule out nonspecific antibody binding and autofluorescence, quadrants were set according to isotype controls. Positive and negative gates for each molecule were also verified on the basis of “fluorescence-minus-one” controls.

### 2.8. Percentages of IL-10- or LAP TGF-*β*1-Producing Treg Cells

Purified CD4^+^ T-cells from patients and control groups were cultured in complete RPMI 1640 media and stimulated with PMA (25 ng/mL), ionomycin (1 *μ*g/mL), and brefeldin A (10 *μ*g/mL) for 5 h. Cell stimulation was confirmed by membrane expression of CD69 molecule using an FITC anti-CD69 antibody. After stimulation, LAP TGF-*β* surface expression was detected using PE-LAP TGF-*β*1 monoclonal antibody (TW4-6H10 clone, BioLegend) or intracellular IL-10 using PE anti-IL-10 monoclonal antibody (JES3-19F1 clone, BioLegend). The cells were analyzed by flow cytometry. The percentage of positive cells, the MFI, and the iMFI was determined.

Data acquisition was performed using FACSCanto II (Becton Dickinson). Flow cytometric analysis was conducted using FlowJo software (TreeStar Inc., Ashland, OR, USA).

### 2.9. Statistical Analysis

All values are expressed as the mean ± SEM. The Mann-Whitney *U* test and Kruskall-Wallis test followed by Dunn's multiple comparison tests were used for a significance level of 0.05. Statistical analyses were performed with Graph Pad Prism software version 5.0 (La Jolla, CA, USA).

## 3. Results

### 3.1. Quantification of Cytokines in Control Groups and in Lung Adenocarcinoma Patients

**Figure 1:**
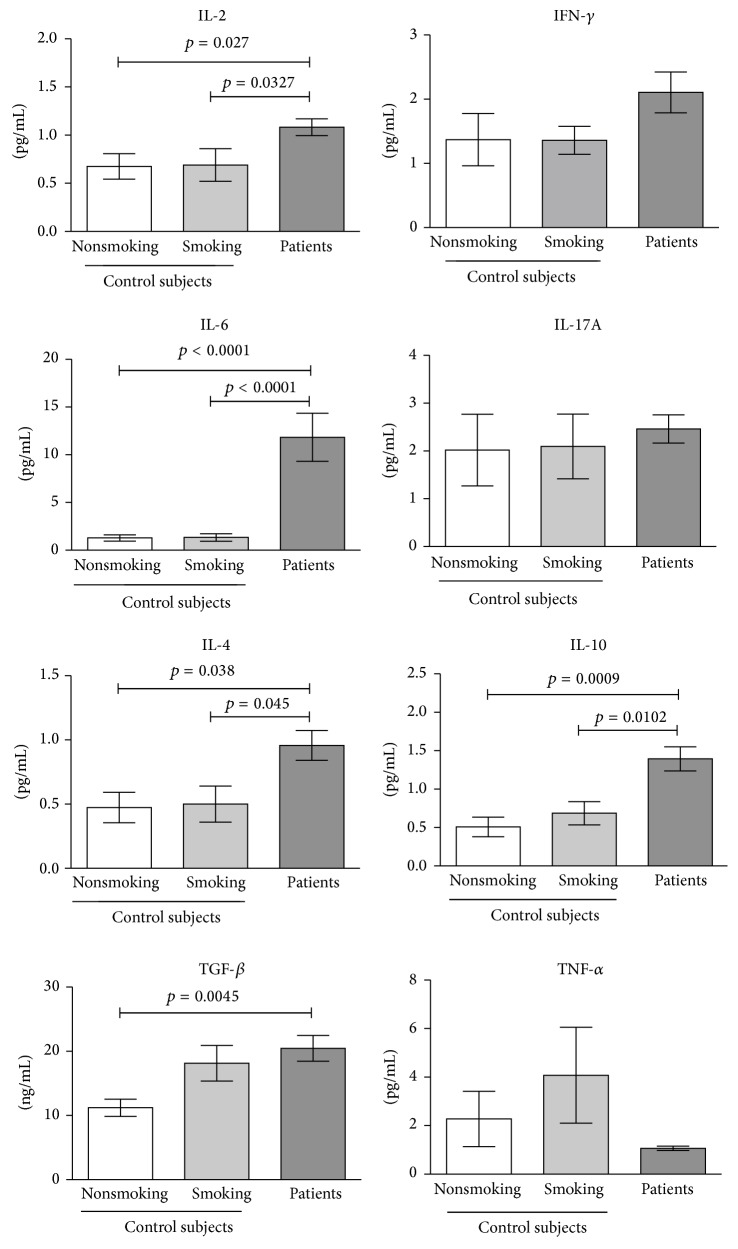
Plasma levels of IL-2, IL-4, IL-6, IL-10, IL-17A, TNF-*α*, IFN-*γ*, and TGF-*β*1 cytokines in lung adenocarcinoma patients and smoking and nonsmoking control subjects. Plasma concentrations of IL-2, IL-4, IL-6, IL-10, IL-17A, TNF-*α*, and IFN-*γ* were measured simultaneously using a cytometric bead array. TGF-*β*1 was measured by ELISA. The results are reported as mean ± SEM.

Cytokines associated with Treg cells or Th17 cells were quantified in the plasma of the control groups and advanced-stage lung adenocarcinoma patients. In the smoking and nonsmoking groups, the plasma concentrations of IL-2, IFN-*γ*, IL-6, IL-17A, IL-4, and IL-10 were similar. A tendency to increase the levels of TGF-*β*1 and TNF-*α* was found in the smoking group. See Figure 1.

In the lung adenocarcinoma patients, significant increases in TGF-*β*1 were detected compared with nonsmoking subjects. Significant increases in IL-2, IL-4, IL-6, and IL-10 cytokines were found compared with the smoking and nonsmoking groups. Plasma concentrations of IFN-*γ* tended to increase, whereas TNF-*α* tended to decrease compared with both control groups. See Figure 1.

### 3.2. Percentages of IL-17A-Producing CD4^+^ T-Cells in Control Groups and Lung Adenocarcinoma Patients

IL-17A-producing CD4^+^ T-cells were quantified from stimulated PBMCs. It is known that Th1 cells produce IL-17 in addition to IFN-*γ*. Thus, IL-17A/IFN-*γ* double-positive cells were detected to rigorously identify IL-17-producing CD4^+^ (Th17) T-cells.

**Figure 2:**
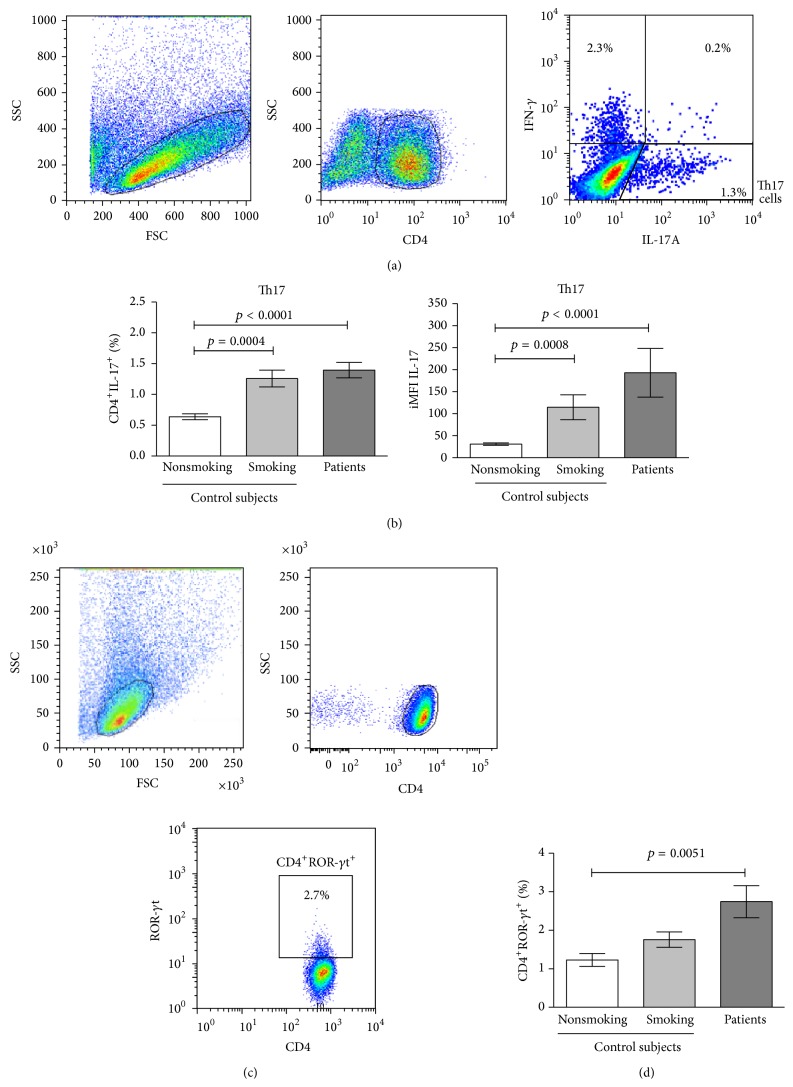
Percentages of Th17 cells were identified according the membrane expression of ROR-*γ*t or intracellular IL-17 in lung adenocarcinoma patients and smoking and nonsmoking control subjects. (a) Distribution of stimulated PBMCs according to forward scatter (FSC) and sideward scatter (SSC) dot plot. A gating was set for CD4^+^ T-cells. From gated CD4^+^ T-cells, cells producing IL-17, IFN-*γ*, or both cytokines were detected. A representative cytometric analysis from a lung adenocarcinoma patient is shown. (b) Percentages and expression levels (measure by iMFI values) of IL-17 from CD4^+^IL-17^+^ (Th17) T-cells are shown and compared among the studied groups. The results are reported as mean ± SEM. (c) Distribution of purified CD4^+^ T-cell in a FSC and SSC dot plot. A gating was set for CD4^+^ T-cells. From gated CD4^+^ T-cells, the percentage of ROR-*γ*t^+^ cells was detected. A representative cytometric analysis from a lung adenocarcinoma patient is shown. (d) Percentages of CD4^+^ROR-*γ*t^+^ T-cells from lung adenocarcinoma patients and smoking and nonsmoking control subjects are shown. The results are reported as mean ± SEM.

The percentage of Th17 cells in the smoking group significantly increased 1.95-fold compared with the percentage in the nonsmoking group. In lung adenocarcinoma patients, the percentage of Th17 cells was similar to the smoking group and significantly increased by 2.2-fold compared with the percentage in the nonsmoking group. See Figures 2(a) and 2(b).

In the smoking group, the expression of IL-17A, as detected by the MFI value, showed a tendency to increase compared with the nonsmoking group. In lung adenocarcinoma patients, significant increases were found in the MFI values of IL-17A compared with the nonsmoking group (data not shown). The iMFI reflects the total functional response in terms of quality (MFI) and magnitude (percentage) as previously described [26]. A significant increase of the iMFI values of IL-17A was detected in the smoking group compared with the nonsmoking group. In summary, Th17 cells from lung adenocarcinoma patients, compared with the control groups, expressed high levels of IL-17A. These results indicate that Th17 cells from the studied groups are functional. Moreover, the Th17 cells from lung adenocarcinoma patients produced the highest levels of IL-17A. See Figure 2(b).

### 3.3. Percentages of ROR-*γ*t CD4^+^ T-Cells in Control Groups and Lung Adenocarcinoma Patients

We determined the proportions of Th17 cells in peripheral blood identifying this subset as CD4^+^ROR-*γ*t^+^ T-cells. In nonsmoking and smoking control subjects, similar percentages of CD4^+^ROR-*γ*t^+^ T-cells (1.22 versus 1.75%) were detected. In lung adenocarcinoma patients, the percentage of CD4^+^ROR-*γ*t^+^ T-cells was 2.75%. A significant increase of 2.2-fold was detected in the percentage of CD4^+^ROR-*γ*t^+^ T-cells in lung adenocarcinoma patients compared with nonsmoking subjects. See Figures 2(c) and 2(d). Interestingly, in lung adenocarcinoma patients, percentages of ROR-*γ*t^+^ CD4^+^ T-cells were higher compared to the percentages of IL-17A-producing CD4^+^ T-cells.

For the level of expression of ROR-*γ*t, the MFI values were similar in the lung adenocarcinoma and control groups. The smoking group showed a tendency to increase iMFI values compared with the nonsmoking group. Lung adenocarcinoma patients showed increases in this parameter compared with the nonsmoking and smoking groups (data not shown). However, no significant differences were found when the three groups were compared.

### 3.4. Percentages of Treg Cells in Control Groups and Lung Adenocarcinoma Patients

**Figure 3:**
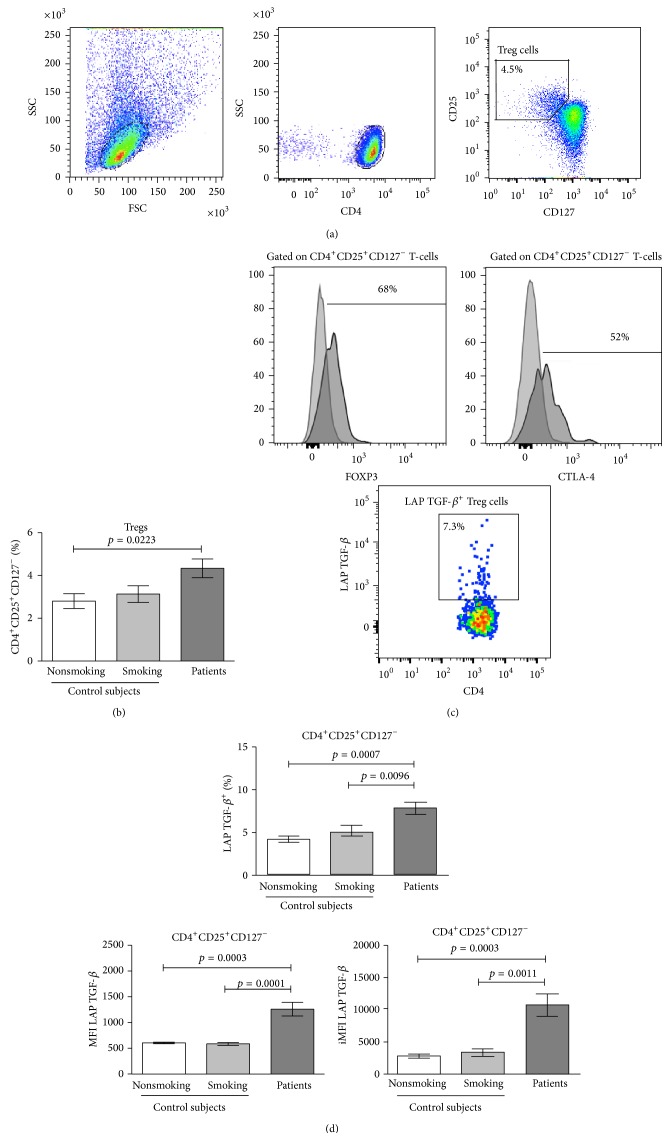
Percentages of Treg cells phenotyped as CD4^+^CD25^+^CD127^−^ and percentages and expression levels of LAP TGF-*β* in Treg cells from lung adenocarcinoma patients and smoking and nonsmoking subjects. (a) Distribution of purified CD4^+^ T-cell in a forward scatter (FSC) and sideward scatter (SSC) dot plot. A gating was set for CD4^+^ T-cells. From gated CD4^+^ T-cells, the percentage of CD25^+^CD127^−^ cells was detected. A representative cytometric analysis from a lung adenocarcinoma patient is shown. (b) Percentages of Treg cells from lung adenocarcinoma patients and smoking and nonsmoking control subjects are shown. The results are reported as mean ± SEM. (c) The histograms indicate the percentages of gated unstimulated Treg cells expressing FOXP3 or CTLA-4. From stimulated CD4^+^ T-cells, the percentage of LAP TGF-*β* ^+^ cells from Treg cells was detected. A representative cytometric analysis from a lung adenocarcinoma patient is shown. (d) Percentages and expression levels (measure by MFI and iMFI values) of LAP TGF-*β* from CD4^+^CD25^+^CD127^−^ T-cells are shown and compared among the studied groups. The results are reported as mean ± SEM.

We analyzed the frequency of Treg cells identified as CD4^+^CD25^+^CD127^−^ T-cells. In the present study, similar percentages of CD4^+^CD25^+^CD127^−^ Treg cells were detected in the smoking and nonsmoking subjects. A significant increase in the percentage of Treg cells was detected in lung adenocarcinoma patients compared with nonsmoking subjects. See Figures 3(a) and 3(b). FOXP3 has been shown to be inversely correlated with the CD127 molecule [15]. Our results show that approximately 65–75% of CD4^+^CD25^+^CD127^−^ Treg cells expressed FOXP3 in the studied groups. See Figure 3(c).

### 3.5. Treg Cells Producing IL-10 or Expressing CTLA-4

The expression of IL-10 and CTLA-4, molecules involved in the suppressor activity of Treg cells, were studied. CD4^+^CD25^+^CD127^−^ Treg cells from the control groups and lung adenocarcinoma patients did not produce IL-10 (data not shown). Approximately 50–60% of the CD4^+^CD25^+^CD127^−^ Treg cells expressed CTLA-4 in lung adenocarcinoma patients and the control groups. See Figure 3(c). In addition, CTLA-4 expression (determined by the MFI and iMFI values) was similar among all groups (data not shown). Thus, our results show that CD4^+^CD25^+^CD127^−^ Treg cells from the smoking and nonsmoking groups and from lung adenocarcinoma patients did not mediate suppressor activity by IL-10 production; nevertheless, CTLA-4 may be involved in the suppressor function.

### 3.6. LAP TGF-*β*1 in Treg Cells from Control Groups and Lung Adenocarcinoma Patients

From among the CD4^+^CD25^+^CD127^−^ T-cell population, LAP TGF-*β*1-producing cells in control groups and lung adenocarcinoma patients were quantified and compared. No significant differences between the control groups were found. However, a significant increase of 1.4-fold to 1.75-fold was detected in the percentages of CD4^+^CD25^+^CD127^−^LAP TGF-*β*1^+^ Treg cells in lung adenocarcinoma patients compared with the corresponding population in the smoking and nonsmoking control groups. See Figures 3(c) and 3(d).

When the expression levels of LAP TGF-*β*1 in the CD4^+^CD25^+^CD127^−^ Treg cells from the groups were analyzed, similar MFI values were found in the smoking and nonsmoking groups. In contrast, the expression level of LAP TGF-*β*1 significantly increased more than 2-fold in lung adenocarcinoma patients in comparison with the smoking and nonsmoking groups. See Figure 3(d). With respect to iMFI values for LAP TGF-*β*1, lung adenocarcinoma patients showed an increase in the expression of LAP TGF-*β*1 that was 3.25-fold and 3.8-fold greater than the expression in the smoking and nonsmoking groups, respectively. See Figure 3(d).

### 3.7. Balance between IL-17-Producing CD4^+^ T-Cells and LAP TGF-*β*1^+^ Treg Cells

**Figure 4:**
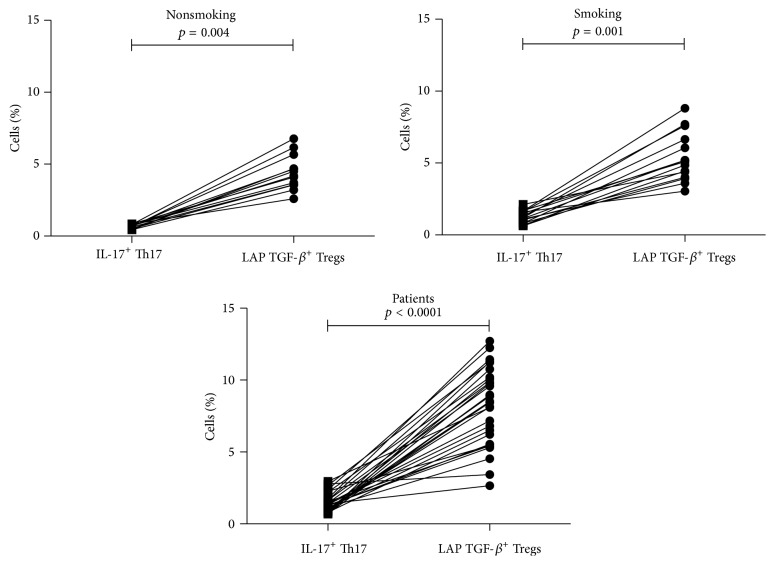
Relationship between IL-17-producing CD4^+^ T-cells (Th17 cells) and LAP TGF-*β* ^+^ Treg cells. Percentages of IL-17-producing CD4^+^ T-cells (Th17 cells) and percentages of LAP TGF-*β* from CD4^+^CD25^+^CD127^−^ Treg cells are shown and compared in lung adenocarcinoma patients and smoking and nonsmoking subjects.

The relationship between IL-17-producing CD4^+^ T-cells (Th17) and CD4^+^CD25^+^CD127^−^LAP TGF-*β*1^+^ Treg cells was explored in each group studied. In all groups, the percentage of LAP TGF-*β*1^+^ Treg cells was higher than the percentage of IL-17^+^ Th17 cells. See Figure 4. To determinate the balance between Th17 and Treg cells the Th17/Treg ratio was calculated. The Th17/Treg ratios were 0.1587 ± 0.016, 0.2527 ± 0.03, and 0.2122 ± 0.03 in nonsmoking subjects, smoking subjects, and patients, respectively. These results indicate that, in smoking subjects, cigarette smoke promotes an inflammation and this is reflected by the increase of IL-17-producing CD4^+^ T-cells, whereas, in lung adenocarcinoma patients, who were classified as heavy smokers, the tumor might modify the balance to immunosuppression, mediated by the increase in the percentage of the LAP TGF-*β*1^+^ subset of CD4^+^CD25^+^CD127^−^ Treg cells.

In summary, our results show that, at the systemic level, the percentage of the LAP TGF-*β*1^+^ subset of CD4^+^CD25^+^CD127^−^ Treg cells was increased in advanced-stage lung adenocarcinoma patients. Overexpression of LAP TGF-*β*1, associated with this Treg subset, might suppress antitumoral responses.

## 4. Discussion

Few studies have compared the cytokine profile and Th17/Treg balance between healthy subjects and healthy heavy smokers [27]. Thus, we studied the impact of the inflammatory response on cytokine concentrations and on the percentages of Th17 and Treg cells at the systemic level. Even when no differences were found in the cytokine profile between smoking and nonsmoking subjects, our results indicate that smoking is promoting inflammation by increasing IL-17-producing Th17 cells. These cells might promote an inflammatory response; however, as smoking control subjects showed no sign of smoking-related respiratory diseases and no increases of IL-6 and IL-17A were found, the slight increase in TGF-*β* levels in the smoking group may be participating in regulation of the inflammatory response. This mechanism would prevent the development of a pathological condition and maintain a transient inflammatory state in which the recovery of the homeostasis is still possible.

In lung cancer patients, previous studies have evaluated pro- and anti-inflammatory systemic cytokines and Th17 and Treg cells [18, 19, 23, 28–30]. In those studies, data were compared with healthy control subjects, which included both smoking and nonsmoking subjects. As smoking may affect systemic cytokines and the relationship of Th17 and Treg cells; in this study, we decided to analyze the data obtained from lung adenocarcinoma patients and compare these data with those of smoking subjects. This knowledge can show us whether chronic inflammation or suppression predominates at the systemic level in lung adenocarcinoma patients.

Our results showed higher levels of proinflammatory (IL-2 and IL-6) and anti-inflammatory (IL-4 and IL-10) cytokines in lung adenocarcinoma patients compared with smoking subjects. With respect to IL-2, a previous report indicates that this cytokine increases in NSCLC patients at local and systemic levels [31], and our data agree with this report. IL-6 has primarily been associated with the inflammatory response [32]. Increases in this cytokine have been reported in lung cancer patients and have been associated with tumor progression and poor prognosis [33, 34]. IL-6 produced by lung tumors might explain the increase in plasma concentrations detected in lung adenocarcinoma patients. The increased production of IL-6 and TGF-*β* could promote the differentiation of Th17 cells to maintain a chronic inflammatory state.

With respect to Treg cells, Carpagnano et al. proposed that IL-2 in presence of TGF-*β* might promote the generation and differentiation of Treg cells in lung cancer [31]. The increased levels of these cytokines detected in our study might be supporting this phenomenon. In addition, IL-4 and IL-10 may also participate in the differentiation process to Treg cells, as higher levels of these cytokines were also detected in our study.

In NSCLC, increases in Treg cells, immunophenotyped as CD4^+^CD25^+^ or CD4^+^CD25^+^FOXP3^+^, have been previously reported [18, 19, 28, 29]. In those studies, no distinction was made between activating T-cells, which temporarily express CD25 and FOXP3 and Treg cells. In recent studies, a more strict characterization of Treg cells has been achieved [23]. In some of these studies, the suppressor activity of Treg cells has been evaluated by examining the expression of IL-10, TGF-*β*, and/or CTLA-4 [19, 29, 35]. We found that, in lung adenocarcinoma patients, the suppressor function of Treg cells is not mediated by IL-10. However LAP TGF-*β*, a membrane molecule with inhibitory activity, is involved. Even though the effector function of the LAP TGF-*β* subset of the CD4^+^CD25^+^CD127^−^ Treg cells was not directly evaluated in our study, Mahalingam et al. and Scurr et al. reported that peripheral blood and tumor-infiltrating LAP^+^ Treg cells exhibit potent suppressive activity in colorectal cancer [20, 21]. Thus, the LAP^+^ Treg cells detected in our study may be participating in the immunosuppression observed in lung cancer patients.

To clarify to what extent smoking-associated inflammation contributes to pathogenesis of lung adenocarcinoma, we analyzed Th17 cells. Some groups have reported that Th17 cells (detected as IL-17-producing CD4^+^ T-cells) are increased in peripheral blood of lung cancer patients [24]; our data agree with these previous reports. As ROR-*γ*t is the master transcription factor of Th17 cells, we analyzed CD4^+^ T-cells expressing this marker. We previously reported, in malignant pleural effusion from lung cancer patients, similar percentages of CD4^+^ROR-*γ*t^+^ T-cells and IL-17-producing Th17 cells [36]. In peripheral blood of lung adenocarcinoma patients, higher proportions of CD4^+^ROR-*γ*t^+^ T-cells compared with the IL-17-producing Th17 cells were found. These results might be related to the anatomical compartment rather than to the underlying pathology; another possibility is that the Th17 cells in peripheral blood are dysfunctional. The increase in Treg cells, acting by LAP TGF-*β* mechanism, might inhibit the effector function of Th17 cells. Further studies are required to study this possibility.

Th17 and Treg cells have opposite functions, whereas Th17 cells promote inflammation, Treg cells suppress immune response. In tumors associated with chronic infections, such as gastric cancer, the balance is favored toward to Th17 cells [37, 38]. In patients with NSCLC, Li et al. showed that FOXP3^+^ Treg cells and Th17 cells are positively correlated [24]; however, no distinction between Treg cells and activated T-cells, which transiently express FOXP3, was made. Recently, Zhao et al. found an inverse correlation between Treg cells and Th17 cells in peripheral blood of NSCLC patients, attributing this event to reduction of Th17 cells [23]. In that study, Th17 cells were identified as CD3^+^CD8^−^IL-17^+^ cells; perhaps, the presence of NKT cells affected the accuracy of IL-17^+^ cells quantification. Even though an inflammatory process occurs in smoking subjects, as evidenced by an increased Th17/Treg ratio, our data indicate that in lung adenocarcinoma patients, this balance is reverted favoring increases of Treg cells at the systemic level. Maybe the array of immunosuppressive cytokines that are increased in lung adenocarcinoma patients supports the presence of Treg cells.

In conclusion, as similar increases in Th17 cells were found at the systemic level in smoking subjects and lung adenocarcinoma patients, smoking rather than the tumor caused inflammation. In lung adenocarcinoma patients, compared with smoking subjects, the concentrations of IL-2, IL-4, IL-6, and IL-10 were increased. A higher percentage of CD4^+^CD25^+^CD127^−^LAP TGF-*β* ^+^ Treg cells were found; this subset showed higher levels of LAP TGF-*β*, with respect to the corresponding subset from smoking and nonsmoking subjects.

This knowledge should lead to the development of immunotherapies that inhibit the suppressor activity mediated by LAP TGF-*β* from CD4^+^CD25^+^CD127^−^ Treg cells. This approach alone, or in combination with immunotherapeutic agents targeting the immune checkpoints, would promote the reactivity of immune cells against lung adenocarcinoma cells to increase patient's overall survival rates.
